# Toll-like receptor 2: therapeutic target for gastric carcinogenesis

**DOI:** 10.18632/oncotarget.735

**Published:** 2012-11-06

**Authors:** William McCormack, Masanobu Oshima, Patrick Tan, Brendan J. Jenkins

**Affiliations:** OPSONA Therapeutics Ltd, Dublin, Ireland; Division of Genetics, Cancer Research Institute, Kanazawa University, Kanazawa, Japan; Cancer and Stem Cell Biology, Duke-NUS Graduate Medical School, Singapore; Centre for Innate Immunity and Infectious Diseases, Monash Institute of Medical Research, Monash University, Clayton, Victoria, Australia

Gastric cancer is the second most lethal cancer world-wide, and has a poor overall 5-year survival rate of <25% which is largely due to both late detection of this aggressive disease and the limited effectiveness of current treatment options. Surgery alone can only “cure” a small proportion of patients with locally-invasive advanced gastric cancer, and while adjuvant perioperative chemotherapy (e.g. epirubicin, cisplatin, fluorouracil) reduces tumour sizes and improve the 5-year survival rate [1], patient outcomes are largely marginal and often associated with tumour re-occurrence. Moreover, the need for early detection (i.e. screening) and treatment strategies to improve patient survival rates is highlighted by the disappointing results of phase II/III clinical trials worldwide aimed at investigating the safety and efficacy of new drugs for advanced local (resectable) or metastatic gastric cancer.

Gastric cancer represents an increasing number of cancers, including colon, liver, lung and pancreatic, whereby disease pathogenesis is intimately linked with chronic inflammation (in the case of the stomach, gastritis). It is therefore perhaps not surprising that over recent years there has been an explosion in research to identify key regulators of the immune system with oncogenic potential that promote the transition from a chronic inflammatory state to one of carcinogenesis. In this respect, much attention has focussed on pathogen recognition receptors, in particular members of the Toll-like receptor (TLR) family which act as critical sensors of the immune system to trigger the inflammatory response to many microbial (i.e. viral, bacterial, fungal) insults [2]. Support for a role of TLRs in gastro-intestinal carcinogenesis has come from a spate of clinical data indicating increased expression of TLRs in patient tumour biopsies or precursory inflamed lesions [3], as well as animal disease models in which mice lacking the key TLR signaling adaptor MyD88 are protected from experimentally-induced colorectal cancer [4]. Despite such observations, a causal role for specific TLRs in the molecular pathogenesis of gastro-intestinal malignancies, and more specifically gastric cancer, is lacking.

A recent study by Tye and colleagues has paved the way for new insights into this issue, based on the discovery that the specific and augmented expression of the *TLR2* gene in tumours of advanced gastric cancer patients is associated with poor overall survival [5]. Furthermore, genetic and antibody-mediated therapeutic (OPN301, developed by Opsona Therapeutics), targeting of TLR2 in a pre-clinical gastric cancer mouse model displaying elevated gastric TLR2 expression levels dramatically suppressed gastric tumour growth independent of inflammation, thus uncovering a novel growth regulatory role for TLR2 on the gastric epithelium [5].

The identification of TLR2 as a new gastric cancer gene now paves the way for several key avenues of research. For example, pinpointing specific molecular pathways acting downstream of TLR2 critically responsible for gastric carcinogenesis is an important question, which could be potentially addressed through the identification of gene signatures triggered by TLR2 activation in gastric epithelial tumour cells, and mapping these TLR2 signatures to databases of other known pathway activation profiles. The availability of a robust disease-associated TLR2 signature could also facilitate patient stratification efforts in selecting those specific gastric cancer patients that are most likely to respond to a TLR2-targeting therapy. We also propose that future research efforts be directed towards examining the genomes of primary gastric tumours to identify somatic alterations in TLR2 (either mutation or gene amplification) that might cause hyper-activation of TLR2, analogous to the epidermal growth factor receptor in lung cancer.

In addition to its newfound role in gastric cancer, TLR2 has been implicated in numerous other inflammatory diseases and cancers, such as lung and pancreatic cancer, arthritis, and ischemia reperfusion (I/R) injury, which collectively make TLR2 an attractive therapeutic target. In this regard, the murine monoclonal antibody OPN301 used by Tye and colleagues to effectively suppress gastric tumour growth is also efficacious in pre-clinical models of kidney transplantation, cardiac I/R injury and pancreatic cancer [6]. Based on these observations, a humanised version of this antibody (OPN305, Opsona Therapeutics) is in late stage clinical development, and will enter a Phase II clinical study in early 2013 to evaluate its safety, tolerability and efficacy in renal transplant patients at high risk of Delayed Graft Function, as the first clinical target indication for OPN305.

We do note, however, the existence of additional approaches to inhibit TLR2 activity *in vitro*, including short interfering RNA (siRNA), small molecule inhibitors identified in cell based screening assays, and peptide mimetics that prevent ligand-induced receptor signalling of the intracellular domain of TLR2. While the anti-tumour efficacy of such strategies *in vivo* remains to be explored, a recent *in vivo* study successfully used TLR2-specific siRNA to block tumourigenesis in a xenograft model of liver cancer [7].

Our recent study suggests that at least in the context of gastric cancer, TLR2 antagonists can ameliorate tumour growth by acting directly on the tumour cells to promote apoptosis and suppress proliferation [5]. However, it is important to consider that TLR agonists (including those for TLR2) can induce adaptive immune responses against cancer cells, which supports the application of TLR agonists in cancer vaccine therapy [8]. Therefore, whether TLR2 antagonists will serve as efficacious therapies against other tumour types, especially those where TLR2 may transmit an anti-tumour immune response will require stringent evaluation in pre-clinical animal-based cancer models.
